# Steroid refractory granulomatous mastitis treated by top surgery: A case report

**DOI:** 10.1097/MD.0000000000030730

**Published:** 2022-10-28

**Authors:** Ya-Di Lu, Yen-Chen Yu, Dun-Hao Chang

**Affiliations:** a Department of Medical Education, Taipei Veterans General Hospital, Taipei City, Taiwan; b Division of Plastic and Aesthetic Surgery, Department of Surgery, Far Eastern Memorial Hospital, Taipei City, Taiwan; c Department of Information Management, Yuan Ze University, Taoyuan, Taiwan; d School of Medicine, National Yang Ming Chiao Tung University, Taipei, Taiwan.

**Keywords:** double incision, granulomatous mastitis, mastectomy, steroid

## Abstract

Granulomatous mastitis (GM) is a rare inflammatory disease and the presentation mimics infectious mastitis or breast cancer. The disease usually develops at the unilateral breast in women with breast-feeding history at their child-bearing age. Systemic steroids had been proposed as the first-line treatment, the combination of surgery was also recommended for complicated disease. However, recurrence might still happen in some rare cases. Few studies have addressed the management of such difficult situations.

**Patient concerns:**

We report the case of a 33-year-old androgynous and nulliparous woman who initially presented left breast erythematous swelling and was treated as infectious mastitis with debridement and antibiotics.

**Diagnosis:**

After wider excision for pathology, the diagnosis of GM was confirmed.

**Interventions:**

Steroids combined with methotrexate were prescribed. However, the symptoms only subsided temporarily and progressed to the contralateral side within 3 months. She finally underwent double-incision mastectomy and free nipple grafting.

**Outcomes:**

The surgery was completed uneventfully, and she had a satisfactory result with no more recurrence at the 6-month follow-up.

**Lesson:**

This GM case with the refractory treatment courses brought out the importance of surgical resection and was the first case report of treating GM with top surgery in the literature. Total mastectomy facilitated a highest complete remission rate of GM and may be advantageous for selected patients, especially in cases where steroids are intolerable.

## 1. Introduction

Granulomatous mastitis (GM), first described in 1972 by Kessler and Wallach,^[1]^ is a benign and chronic breast disease. The disease usually occurs in women of reproductive age and is especially observed coupled with a history of breast-feeding. Typical presentation includes unilateral painful mass, abscess, and areolar retraction; and lymphadenopathy has also been reported.^[2]^ Histopathology is the gold standard of GM diagnosis, since laboratory and imaging assessments are not specific.

There is no optimal treatment guideline to date. A systemic review recommended systemic corticosteroid as the first-line treatment, which had a higher response rate than other medication. If response is insufficient, methotrexate or surgical intervention will be recommended.^[3]^

Here, we report the case of an androgynous patient with refractory GM who was finally treated by bilateral double-incision mastectomy with free nipple grafts.

## 2. Case presentation

**Figure 1.**
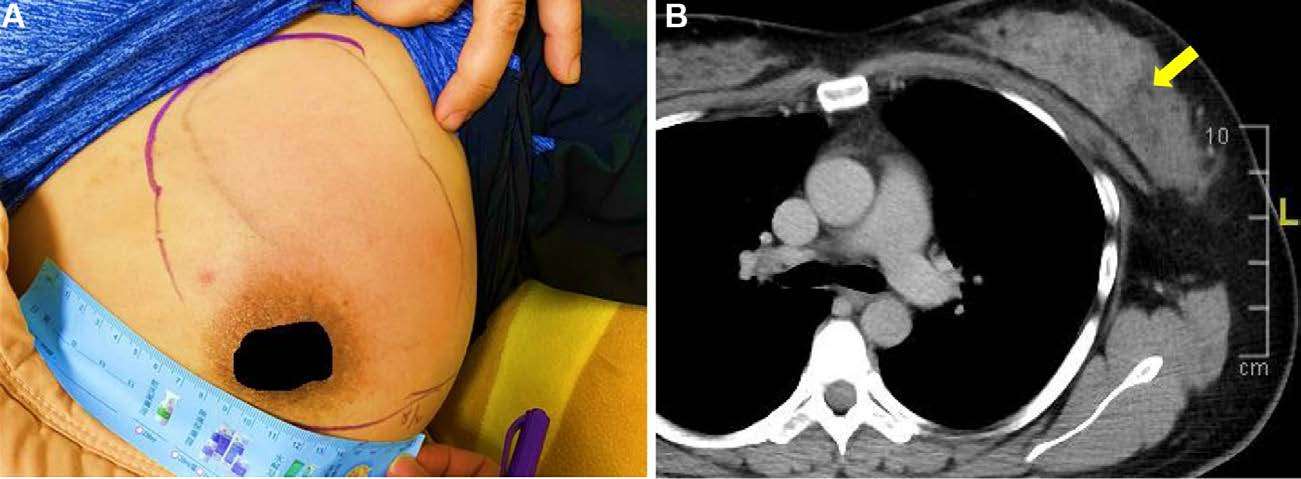
(A) A 33-year-old female patient presented with painful erythematous swelling on the left breast. (B) CT scan demonstrated heterogenous inflammation in the breast gland tissue (indicated by arrow).

A 33-year-old nulliparous female had a history of depression and was taking sulpiride 200 mg twice daily. She presented with painful erythematous swelling at the upper quadrant of her left breast (Fig. 1A). Laboratory examination revealed elevated C-reactive protein (7.925 mg/dL) and leukocytosis (17,960 µL). Ultrasound assessment revealed a 5 × 2 cm heterogeneous hypoechoic mass with BI-RAD 3 classification. Fine needle aspiration revealed no tumor cells. She was treated with empirical antibiotics including amoxicillin/clavulanate and flomoxef but the symptoms didn’t improve. The follow-up CT scan showed left breast tissue swelling with fluid collection and multiple axillary lymphadenopathy (Fig. 1B). Thus, incision and drainage were performed, and green-yellowish abscess was drained. The culture showed small amounts of *Corynebacterium striatum*. However, the painful swelling relapsed within a few days, and she received a second debridement. The histopathology revealed “mastitis,” and the wound cultures became negative. The mass regressed and pain improved thereafter.

**Figure 2.**
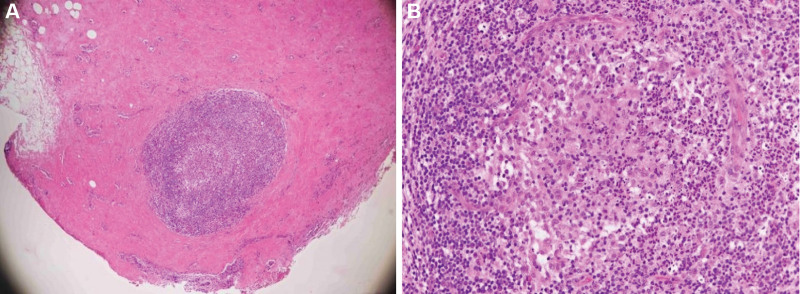
(A) The pathology of granulomatous mastitis. Prominent mixed inflammation in a lobulocentric manner with focal presence of abscesses (H&E, ×40). (B) The Granulomatous inflammation surrounded by lymphocytes, histiocytes and multinucleated giant cells (H&E, ×200).

Nevertheless, the symptoms recurred 2 weeks later. The laboratory examination revealed prolactin 14.20 ng/mL and IgG4 31.90 mg/dL which were all in the normal range. Wider debridement with breast tissue excision was performed. Pathological examination revealed granulomatous inflammation with lymphocytes, histiocytes and multinucleated giant cells infiltrates, confirming the diagnosis of GM. (Fig. 2) Therefore, prednisolone (30 mg twice a day) was prescribed. The symptoms subsided, and she was discharged with the tapered dose of steroid (20 mg/d) and methotrexate (7.5 mg/wk).

**Figure 3.**
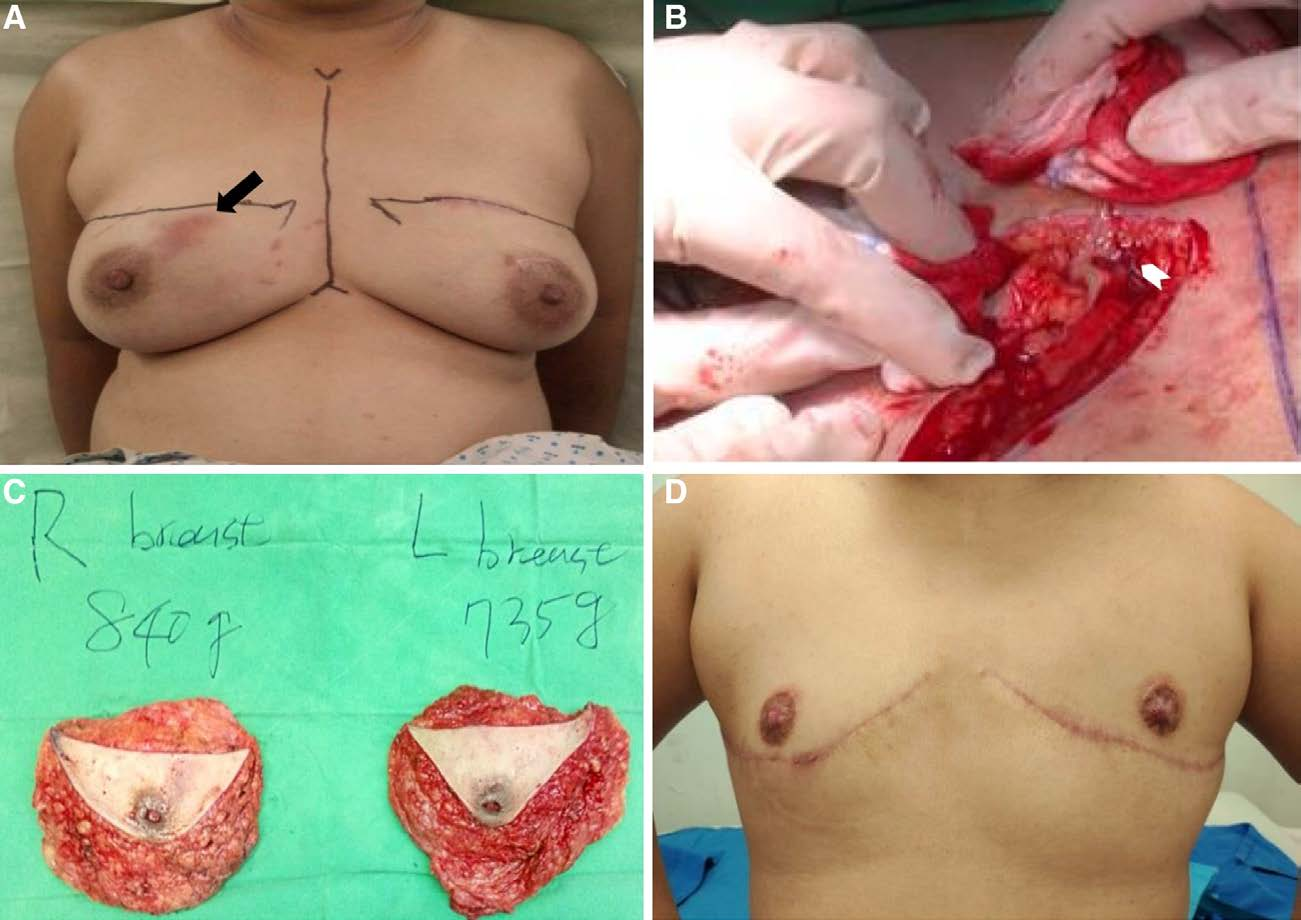
(A) Recurrent erythematous swelling of right breast (black arrow). Preoperative design of double-incision mastectomy was marked. (B) Sloughy purulent granulation tissue was noted during dissection (white dart). (C) The excised specimens. (D) The 3-mo follow-up.

However, 3 months later, erythematous swelling of the right breast developed (Fig. 3). In addition, she also complained of severe side effects of systemic steroids including weight gain and abdominal striae and was also frustrated by this refractory disease. Because she had been in an androgynous life style and used chest binding for more than 10 years, she was desperate for total breast removal. After thorough discussion with the patient and her family, double-incision mastectomy with free nipple grafting was performed. (see Video S1, Supplemental Digital Content, http://links.lww.com/MD/H831 which demonstrates the surgical procedure).

**Figure 4.**
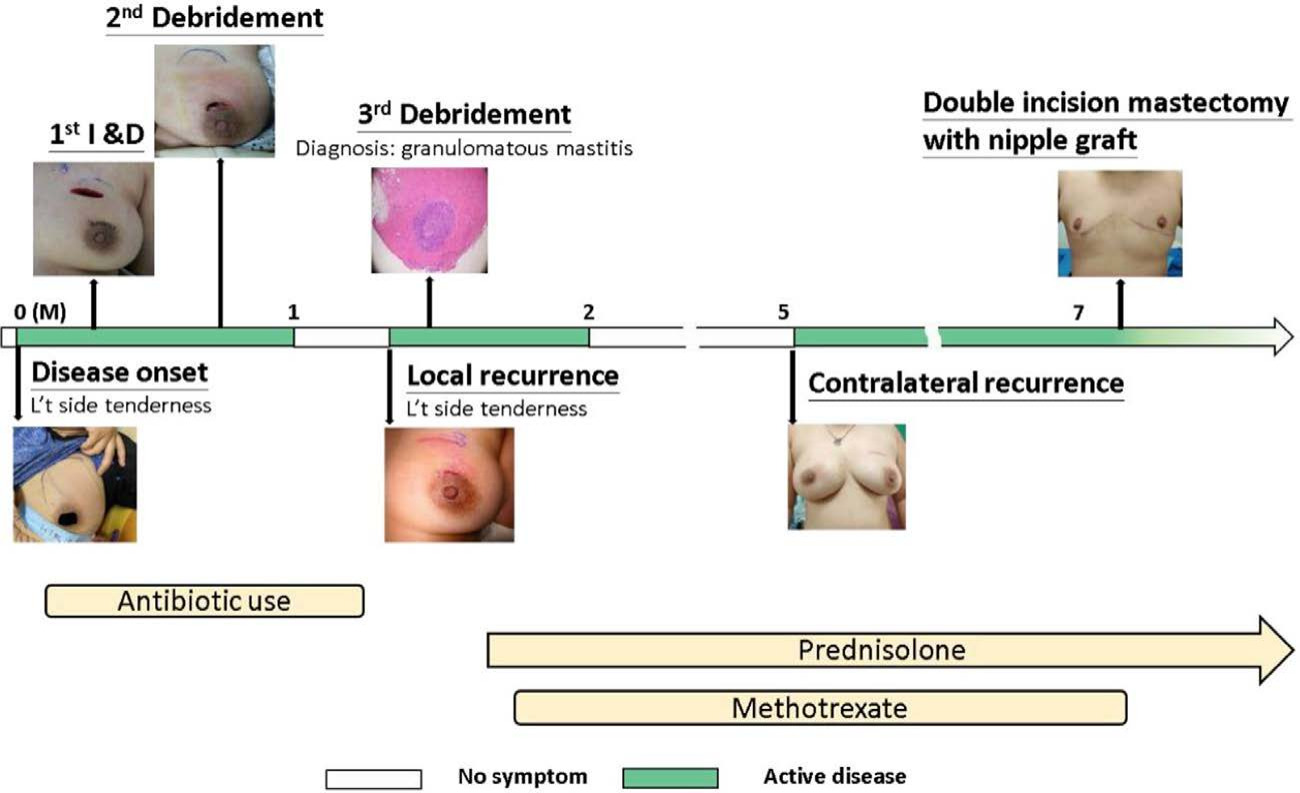
Timeline and the whole course of the granulomatous mastitis patient.

The surgery was completed uneventfully, and she was satisfied with the result. She had no recurrence at the 6-month follow-up and prednisolone was gradually tapered. The whole clinical and treatment course of this case is illustrated in Figure 4.

## 3. Discussion and conclusions

The clinical presentation and radiological findings of GM are similar to infectious mastitis. Some hypothetical etiologies have been proposed, including autoimmune reaction, *Corynebacterium* infection, or secondary hyperprolactinemia provoked by antipsychotic medication. Interestingly, our case had used the antipsychotics without hyperprolactinemia and the culture of *Corynebacterium* was found only once. Therefore, the pathogenesis of our patient was still ambiguous.

Since the manifestations of GM are nonspecific, diagnosis relies on histopathological analysis.^[4,5]^ Core or incisional biopsy is thought to have higher sensitivity than FNA (96% vs 21.1%).^[6]^ In our case, the pathological analysis after the first debridement showed inflammation with poorly-formed granuloma due to the small specimen obtained. The diagnosis of GM was finally made from larger tissue excised during the third operation. Incisional or excisional biopsy may be necessary in such recurrent mastitis.

**Figure 5.**
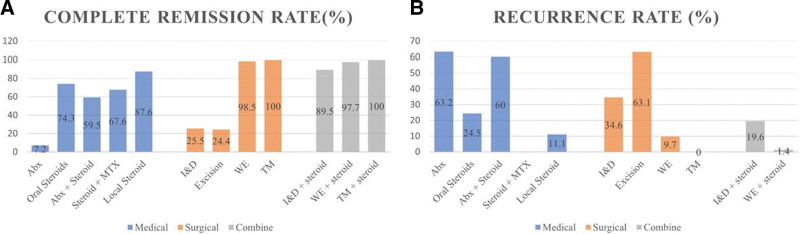
The complete remission rate and recurrence rate of different treatment modalities of granulomatous mastitis. (A) Complete remission rate. (B) Recurrence rate. Abx = antibiotics, I&D = incision and drainage, MTX = methotrexate, TM = total mastectomy, WE = wide excision.

To further assess different treatment approaches for GM and their outcomes, we performed a literature review of recent studies published on PubMed during 2010 to 2021. The outcomes, including complete remission (CR) and recurrence rate, were calculated by data pooling according to different treatment modalities. CR refers to no more pain, or pus formation found in clinical symptom or image study, and recurrence is defined as reappearance of GM symptoms after CR. In total, there were 26 studies with 1124 patients included. The reviewed treatment outcomes of GM are illustrated in Figure 5 (also see Tables S1 and S2, Supplemental Digital Content, http://links.lww.com/MD/H832 which show the summary and detailed information of the literature review).

The antibiotics were usually given because the diagnosis was unclear at the disease onset. However, the antibiotics alone were ineffective with low CR rate (7.2%) and high recurrence rate (63.2%) in our review.

The recommended first-line medical treatment is prednisolone with a dose of 30 mg or up to 1 mg per kilogram per day for at least 2 months.^[7]^ A randomized controlled trial also proved the high dose steroids (50 mg/d × 3 days then gradually tapered to 5 mg/d) had better treatment outcomes than low dose steroids (5 mg/d).^[8]^ Our review showed that the overall CR rate was 74.3% with steroids, but recurrence was reported for some patients (24.5%).

Nevertheless, the side effects of systemic steroids, including hyperglycemia, gastrointestinal problems and weight gain, couldn’t be tolerated in some patients. Therefore, the treatment of steroids given by local injection or topical use had been proposed, demonstrating high CR rate (100%) with low recurrence rate (10%–18%). However, the case numbers are limited in these studies and were confined to uncomplicated GM while those with abscess and fistula were excluded, and more clinical evidence would be required.^[9–11]^

**Figure 6.**
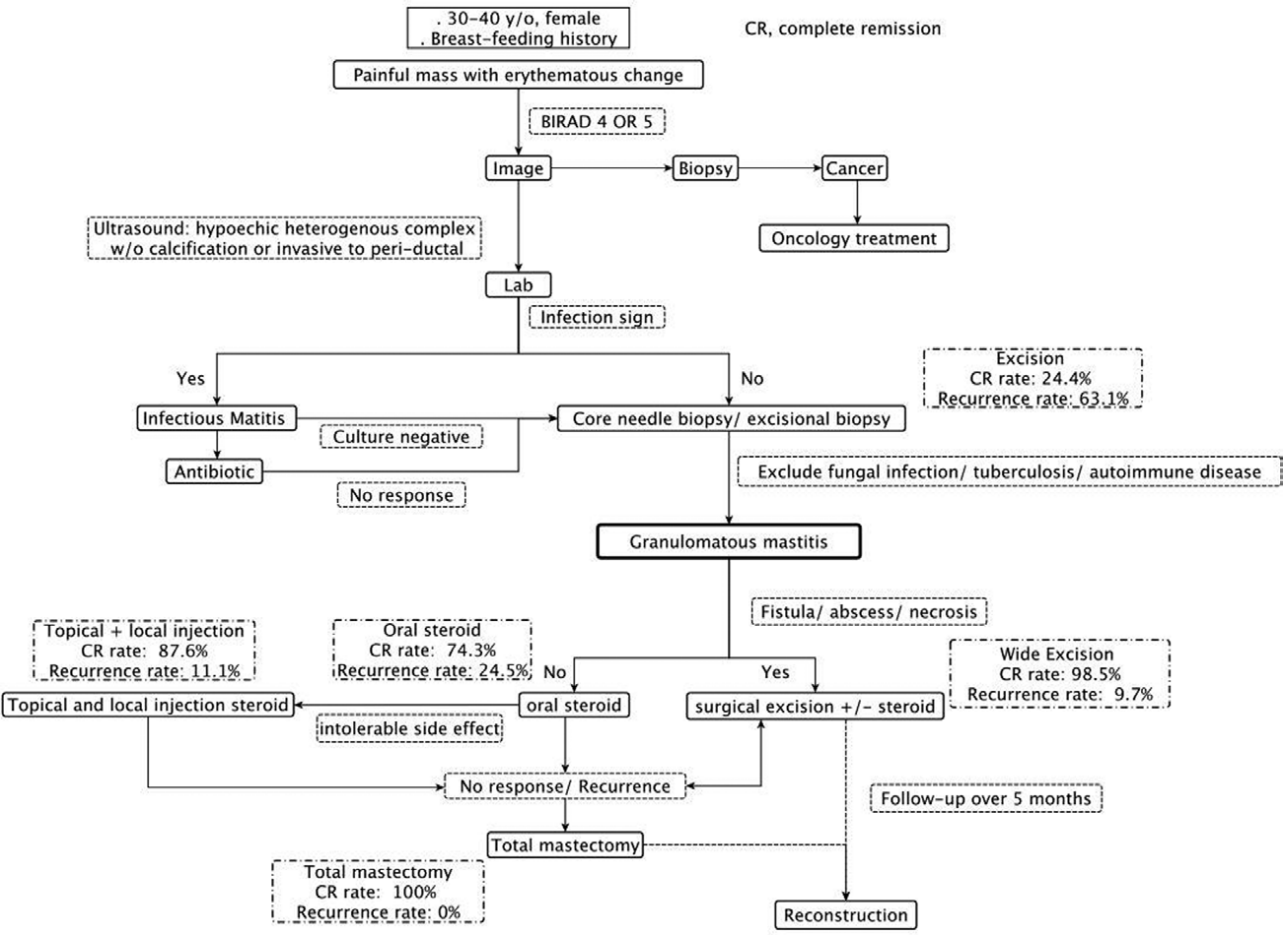
Proposed algorithm of diagnosis and management of granulomatous mastitis.

For patients with complicated or recurrent GM, like our case, the surgical treatments would be usually required. According to our review, we found that the wider the extent of surgical excision, the higher the CR rates were. Although recurrence rates were still around 3.3% to 33.3%, combination with steroids could reduce it to 0% to 10%. Total mastectomy with removal of all the breast tissue could definitely achieve 100% CR and no recurrence. Owing to the lack of life-long follow-up in GM cases, there is still a doubt about the recurrent rate in the patients, who were in remission with medical treatment. Erozgen et al^[12]^ also reported that surgery is a more favorable method than oral steroid use for recurrent or complicated GM with abscess or fistula. Total mastectomy can be considered in selective cases, especially for refractory ones similar to the patient presented here. To the best of our knowledge, the GM patient treated by top surgery has not been described before. Based on the treatment experience of this refractory GM case and the literature review, we proposed a diagnostic and treatment algorithm for future application (Fig. 6).

We have reported here a case of refractory GM involving bilateral breasts, which was successfully treated with double-incision subcutaneous mastectomy and free nipple grafts. We believe that this is the first such report in literature. We recommend wide excision or mastectomy as a treatment option if treatment with steroids is ineffective or results in intolerable side effects.

## Author contributions

Y-DL and D-HC wrote the first draft of the manuscript. D-HC and Y-CY performed the surgery. Y-DL arranged the figures. Y-CY and D-HC reviewed and edited the manuscript. All authors reviewed and edited the manuscript and approved the final version of the manuscript.

**Methodology:** Dun-Hao Chang.

**Supervision:** Dun-Hao Chang.

**Writing – original draft:** Ya-Di Lu, Dun-Hao Chang.

**Writing – review & editing:** Yen-Chen Yu, Dun-Hao Chang.

## Supplementary Material



## References

[1] Kessler E Wolloch Y. Granulomatous mastitis: a lesion clinically simulating carcinoma. Am J Clin Pathol. 1972;58:642–6. 4674439 10.1093/ajcp/58.6.642

[2] Wolfrum A Kümmel S Theuerkauf I Pelz E Reinisch M. Granulomatous mastitis: a therapeutic and diagnostic challenge. Breast Care. 2018;13:413–8. 30800035 10.1159/000495146 PMC6381909

[3] Martinez-Ramos D Simon-Monterde L Suelves-Piqueres C. Idiopathic granulomatous mastitis: a systematic review of 3060 patients. Breast J. 2019;25:1245–50. 31273861 10.1111/tbj.13446

[4] Calis H Karabeyoglu SM. Follow-up of granulomatous mastitis with monitoring versus surgery. Breast Dis. 2017;37:69–72. 28655119 10.3233/BD-160259

[5] Nguyen MH Molland JG Kennedy S Gray TJ Limaye S. Idiopathic granulomatous mastitis: case series and clinical review. Intern Med J. 2021;51:1791–7. 34713960 10.1111/imj.15112

[6] Hovanessian Larsen LJ Peyvandi B Klipfel N Grant E Iyengar G. Granulomatous lobular mastitis: imaging, diagnosis, and treatment. AJR Am J Roentgenol. 2009;193:574–81. 19620458 10.2214/AJR.08.1528

[7] Aghajanzadeh M Hassanzadeh R Alizadeh Sefat S. Granulomatous mastitis: presentations, diagnosis, treatment and outcome in 206 patients from the north of Iran. Breast. 2015;24:456–60. 25935828 10.1016/j.breast.2015.04.003

[8] Montazer M Dadashzadeh M Moosavi Toomatari SE. Comparison of the outcome of low dose and high-dose corticosteroid in the treatment of idiopathic granulomatous mastitis. Asian Pac J Cancer Prev. 2020;21:993–6. 32334460 10.31557/APJCP.2020.21.4.993 PMC7445984

[9] Gunduz Y Altintoprak F Tatli Ayhan L Kivilcim T Celebi F. Effect of topical steroid treatment on idiopathic granulomatous mastitis: clinical and radiologic evaluation. Breast J. 2014;20:586–91. 25228089 10.1111/tbj.12335

[10] Altintoprak F Kivilcim T Yalkin O Uzunoglu Y Kahyaoglu Z Dilek ON. Topical steroids are effective in the treatment of idiopathic granulomatous mastitis. World J Surg. 2015;39:2718–23. 26148520 10.1007/s00268-015-3147-9

[11] Tang A Dominguez DA Edquilang JK Green AJ Khoury AL Godfrey RS. Granulomatous mastitis: comparison of novel treatment of steroid injection and current management. J Surg Res. 2020;254:300–5. 32497924 10.1016/j.jss.2020.04.018

[12] Erozgen F Ersoy YE Akaydin M. Corticosteroid treatment and timing of surgery in idiopathic granulomatous mastitis confusing with breast carcinoma. Breast Cancer Res Treat. 2010;123:447–52. 20625813 10.1007/s10549-010-1041-6

