# Supplementary material for: Steroid refractory granulomatous mastitis treated by top surgery: A case report
Source: Medicine (Baltimore). 2022 Oct 28;101(43):e30730. doi: 10.1097/MD.0000000000030730 (PMC9622708; doi:10.1097/MD.0000000000030730)
Supplement: Supplementary file 2 [file medi-101-e30730-s002.pdf]

Appendix 1. Summary of literature review of granulomatous mastitis treatment

Treatment	Study number	Total patient number	Complete remission rate(%)	Recurrence rate(%)
**Medical**				
Antibiotics	11	371	7.2	63.2
Oral steroids	15	249	74.3	24.5
Abx + steroids	8	248	59.5	60
Steroid +†MTX	5	74	67.6	0
Topical/ local steroid	5	89	87.6	11.1
**Surgical**				
I&D	12	129	25.5	34.6
Excision	11	111	24.4	63.1
Wide excision	10	136	98.5	9.7
Total mastectomy	4	6	100	0
**Steroid combined surgery**				
I&D	3	57	89.5	19.6
Excision	3	22	95	4.8
Wide excision	8	86	97.7	1.4
Total mastectomy	1	1	100	NA
**Overall**	26	1124		

†MTX: methotrexate.

Complete remission (CR) was defined as the eradication of all the inflammatory signs. including resolution of erythema and lump, or fistula with wound total healing.

Recurrence was meant that the aforementioned sign reemerged after the complete remission.

Appendix 2.

Complete remission (CR) was defined as the eradication of all the inflammatory signs. including resolution of erythema and lump, or fistula with wound total healing.

Recurrence was meant that the aforementioned sign reemerged after the complete remission.

Treatment	Study	Total patient number	No. of patient in treatment strategy	Complete remission rate(%)	Recurrence rate(%)	Follow-up
Medical						
Antibiotics	Yau (2010) [1]	11	10	0	NA	18.6m
	Neel (2013) [2]	23	14	28.6	21.4	75m(14m-28years)
	Hur (2013) [3]	50	3	33.3	NA	32m
	Akcan (2014) [4]	74	19	0	NA	3m-170m
	Bouton (2015) [5]	37	22	0	NA	7.4m(0-20m)
	Mahlab-Guri (2015) [6]	17	10	20	0	0.5y-11y
	Yabanoglu (2015) [7]	77	8	0	NA	(16-34m)
	Aghajanzadeh (2015) [8]	206	206	2.9	NA	9-18m
	Freenman (2017) [9]	14	8	12.5	NA	3m(2-24m)
	Bashir (2017) [10]	18	15	0	NA	14.1m(3m-36m)
	Tang (2021) [11]	77	43	30.2	11.8	18 m (1-131 m)
	Overall		371	7.2	63.2	
Oral steroids	Sakurai (2011) [12]	8	5	100	0	40.7m(14-67m)
	Neel (2013) [2]	23	2	50	0	75m(14m-28years)
	Hur (2013) [3]	50	13	30.8	NA	32m
	Pandey (2014) [13]	49	44	79.5	22.7	6-12m
	Karanlik (2014) [14]	60	23	100	30.4	22-78m
	Mahlab-Guri (2015) [6]	17	2	50	0	0.5y-11y
	Sheybani (2015) [15]	22	15	66.7	0	11.9m(6-22m)
	Kiyak (2014) [16]	24	1	0	NA	34.8m(10m-66m)

	Yabanoglu (2015) [7]	77	44	86.4	20.5	(16-34m)
	Bashir (2017) [10]	18	3	0	NA	14.1m(3m-36m)
	Tang (2020) [17]	49	3	0	NA	9m(3m-14m)
	Montazer (2020) [18]	30	30	63.3	15.8	10.14 m - 15.12 m
	Tang (2021) [11]	77	15	80	NA	18 m (1-131 m)
	Shojaee (2021) [19]	87	23	100	39.1	26 m (8-48 m)
	Toktas (2021) [20]	78	26	69.2	NA	13.8 m - 32.3 m
	Overall		249	74.3	24.5	
Abx + steroids	Yau (2010) [1]	11	3	0	NA	18.6m
	Sakurai(2011) [12]	8	3	66.7	0	40.7m(14-67m)
	Neel (2013) [2]	23	8	87.5	71	75m(14m-28years)
	Altintoprak (2015) [21]	28	21	0	NA	37.2m(12-72m)
	Mahlab-Guri (2015) [6]	17	4	0	NA	0.5y-11y
	Aghajanzadeh (2015) [8]	206	200	72.2	NA	9-18m
	Freeman (2017) [9]	14	3	3.3	NA	3m(2-24m)
	Bashir (2017) [10]	18	6	100	66.7	14.1m(3m-36m)
	Overall		248	59.5	60	
Steroid + MTX	Neel (2013) [2]	23	2	0	NA	75m(14m-28years)
	Mahlab-Guri (2015) [6]	17	1	0	0	0.5y-11y
	Sheybani (2015) [15]	22	11	81.6	NA	11.9m(6-22m)
	Aghajanzadeh (2015) [8]	206	56	71.4	NA	9-18m
	Freeman (2017) [9]	14	2	50	NA	3m(2-24m)
	Overall		74	67.6	0	
Topical/ local steroid	Gunduz (2014) [22]	11	11	100	18.2	17m(12-48m)
	Altintoprak (2015) [21]	28	28	100	10.7	37.2m(12-72m)
	Tang (2020) [17]	49	12	100	NA	9m(3m-14m)
	Toktas (2021) [20]	78	32	65.6	NA	13.8 m - 32.3 m
	Toktas (2021) [23]	6	6	100	0	19.5 m

	Overall		89	87.6	11.1	
Surgical						
I&D	Yau (2010) [1]	11	6	100	100	18.6m
	Gurliyik (2012) [24]	19	3	0	NA	20m(6m-75m)
	Neel (2013) [2]	23	6	0	NA	75m(14m-28years)
	Hur (2013) [3]	50	15	33.3	20	32m
	Gunduz (2014) [22]	11	9	0	NA	17m(12-48m)
	Bouton (2015) [5]	37	1	NA	NA	7.4m(0-20m)
	Mahlab-Guri K (2015) [6]	17	4	75	0	0.5y-11y
	Kiyak (2014) [16]	24	9	100	11.1	34.8m(10m-66m)
	Yabanoglu (2015) [7]	77	26	NA	NA	(16-34m)
	Aghajanzadeh (2015) [8]	206	38	0	NA	9-18m
	Calis (2017) [25]	19	3	100	33.3	11m(4m-13m)
	Bashir (2017) [10]	18	9	0	NA	14.1m(3m-36m)
	Overall		129	25.5	34.6	
Excision	Yau (2010) [1]	11	9	66.7	100	18.6m
	Neel (2013) [2]	23	17	23.5	75	75m(14m-28years)
	Pandey (2014) [13]	49	2	100	0	6-12m
	Akcan (2014) [4]	74	6	NA	NA	3-170m
	Bouton (2015) [5]	37	5	100	60	7.4m(0-20m)
	Mahlab-Guri (2015) [6]	17	1	100	0	0.5y-11y
	Yabanoglu (2015) [7]	77	14	NA	NA	16m-34m
	Aghajanzadeh (2015) [8]	206	43	0	NA	9-18m
	Freeman (2017) [9]	14	3	NA	NA	3m(2-24m)
	Calis (2017) [25]	19	1	100	0	11m(4m-13m)
	Bashir (2017) [10]	18	10	NA	NA	14.1m(3m-36m)
	Overall		111	24.4	63.1	
Wide excision	Yau (2011) [1]	11	6	100	33.3	18.6m
	Hur (2013) [3]	50	13	92.3	8	32m
	Akcan (2014) [4]	74	47	NA	NA	3m-170m

	Kiyak (2014) [16]	24	15	100	6.7	34.8m(10m-66m)
	Yabanoglu (2015) [7]	77	31	100	0	(16-34m)
	Elzahaby (2016) [26]	30	30	100	3.3	19m(8-44m)
	Freeman (2017) [9]	14	9	100	11.1	3m(2-24m)
	Bashir (2017) [10]	18	6	100	0	14.1m(3m-36m)
	Tang (2020) [17]	49	9	100	NA	9m(3m-14m)
	Shojaee (2021) [19]	87	17	100	28	26 m (8-48 m)
	Overall		136	98.5	9.7	
Total mastectomy	Yau (2010) [1]	11	1	100	0	18.6m
	Neel (2013) [2]	23	2	100	0	75m(14m-28years)
	Yabanoglu (2015) [7]	77	2	100	0	16m-34m
	Calis (2017) [25]	19	1	100	0	11m(4m-13m)
	Overall		6	100	0	
Oral steroids + surgery						
I&D	Hur (2013) [3]	50	6	50	33	32m
	Mahlab-Guri (2015) [6]	17	4	12.5	0	0.5y-11y
	Shojaee (2021) [19]	87	47	100	19.1	26 m (8-48 m)
	Overall		57	89.5	19.6	
Excision	Gurliyik (2012) [24]	19	19	100	5.3	20m(6m-75m)
	Neel (2013) [2]	23	1	0	NA	75m(14m-28years)
	Akcan (2014) [4]	74	2	100	0	3m-170m
	Overall		22	95	4.8	
Wide excision	Hur (2013) [3]	50	10	80	10	32m
	Pandey (2014) [13]	49	1	100	NA	6-12m
	Akcan (2014) [4]	74	19	100	0	3m-170m
	Karanlik (2014) [14]	60	37	100	0	22-78m
	Aghajanzadeh (2015) [8]	206	11	100	NA	9-18m
	Bashir (2017) [10]	18	1	100	0	14.1m(3m-36m)
	Tang (2020) [17]	49	1	100	NA	9m(3m-14m)

	Montazer (2020) [18]	30	6	100	0	10.14 m - 15.12 m
	Overall		86	97.7	1.4	
Total mastectomy	Freeman (2017) [9]	14	1	100	NA	3m(2m-24m)
	Overall		1	100	NA	

References:

1. Yau FM, Macadam SA, Kuusk U, Nimmo M, Van Laeken N: **The surgical management of granulomatous mastitis**. *Annals of plastic surgery* 2010, **64**(1):9-16.
2. Néel A, Hello M, Cottereau A, Graveleau J, De Faucal P, Costedoat-Chalumeau N, Rondeau-Lutz M, Lavigne C, Chiche L, Hachulla E: **Long-term outcome in idiopathic granulomatous mastitis: a western multicentre study**. *QJM: An International Journal of Medicine* 2013, **106**(5):433-441.
3. Hur SM, Cho DH, Lee SK, Choi M-Y, Bae SY, Koo MY, Kim S, Choe J-H, Kim J-H, Kim JS: **Experience of treatment of patients with granulomatous lobular mastitis**. *Journal of the Korean Surgical Society* 2013, **85**(1):1-6.
4. Akcan A, Öz AB, Dogan S, Akgün H, Akyüz M, Ok E, Gök M, Talih T: **Idiopathic granulomatous mastitis: comparison of wide local excision with or without corticosteroid therapy**. *Breast Care* 2014, **9**(2):111-111.
5. Bouton ME, Jayaram L, O'Neill PJ, Hsu C-H, Komenaka IK: **Management of idiopathic granulomatous mastitis with observation**. *The American Journal of Surgery* 2015, **210**(2):258-262.
6. Mahlab-Guri K, Asher I, Allweis T, Diment J, Sthoeger ZM, Mavor E: **Granulomatous Lobular Mastitis**. *Sat* 2015, **19**:20.
7. Yabanoğlu H, Çolakoğlu T, Belli S, Aytac HO, Bolat FA, Pourbagher A, Tezcaner T, Yildirim S, Haberal M: **A comparative study of conservative versus surgical treatment protocols for 77 patients with idiopathic granulomatous mastitis**. *The Breast Journal* 2015, **21**(4):363-369.
8. Aghajanzadeh M, Hassanzadeh R, Alizadeh Sefat S, Alavi A, Hemmati H, Esmaeili Delshad MS, Emir Alavi C, Rimaz S, Geranmayeh S, Najafi Ashtiani M *et al*: **Granulomatous mastitis: Presentations, diagnosis, treatment and outcome in 206 patients from the north of Iran**. *Breast* 2015, **24**(4):456-460.
9. Freeman C, Xia B, Wilson G, Lewis J, Khan S, Lee S, Lower E, Edwards M, Shaughnessy E: **Idiopathic granulomatous mastitis: a diagnostic and therapeutic challenge**. *The American Journal of Surgery* 2017, **214**(4):701-706.
10. Bashir MU, Ramcharan A, Alothman S, Beaugris S, Khan SA, Sbeih MA, Engdahl R: **The enigma of granulomatous mastitis: A series**. *Breast Dis* 2017, **37**(1):17-20.
11. Tang ELS, Ho CSB, Chan PMY, Chen JJC, Goh MH, Tan EY: **The therapeutic dilemma of idiopathic granulomatous mastitis**. *Ann Acad Med Singap* 2021, **50**(8):598-605.
12. Sakurai K, Fujisaki S, Enomoto K, Amano S, Sugitani M: **Evaluation of follow-up strategies for corticosteroid therapy of idiopathic granulomatous mastitis**. *Surgery today* 2011, **41**(3):333-337.
13. Pandey TS, Mackinnon JC, Bressler L, Millar A, Marcus EE, Ganschow PS: **Idiopathic granulomatous mastitis—a prospective study of 49 women and treatment outcomes with steroid therapy**. *The Breast Journal* 2014, **20**(3):258-266.
14. Karanlik H, Ozgur I, Simsek S, Fathalizadeh A, Tukenmez M, Sahin D, Dursun M, Kurul S: **Can steroids plus surgery become a first-line treatment of idiopathic granulomatous mastitis?** *Breast care* 2014, **9**(5):338-342.
15. Sheybani F, Sarvghad M, Naderi H, Gharib M: **Treatment for and clinical characteristics of granulomatous mastitis**. *Obstetrics & Gynecology* 2015, **125**(4):801-807.
16. Kiyak G, Dumlu EG, Kilinc I, Tokaç M, Akbaba S, Gurer A, Ozkardes AB, Kilic M: **Management of idiopathic granulomatous mastitis: dilemmas in diagnosis and treatment**. *BMC surgery* 2014, **14**(1):1-5.
17. Tang A, Dominguez DA, Edquilang JK, Green AJ, Khoury AL, Godfrey RS: **Granulomatous Mastitis: Comparison of Novel Treatment of Steroid Injection and Current Management**. *J Surg Res* 2020, **254**:300-305.
18. Montazer M, Dadashzadeh M, Moosavi Toomatari SE: **Comparison of the Outcome of Low Dose and High-Dose Corticosteroid in the Treatment of Idiopathic Granulomatous Mastitis**. *Asian Pac J Cancer Prev* 2020, **21**(4):993-996.

19. Shojaee L, Rahmani N, Moradi S, Motamedi A, Godazandeh G: **Idiopathic granulomatous mastitis: challenges of treatment in iranian women**. *BMC Surg* 2021, **21**(1):206.
20. Toktas O, Konca C, Trabulus DC, Soyder A, Koksal H, Karanlik H, Polat AK, Ozbas S, Yormaz S, Isik A: **A novel first-line treatment alternative for noncomplicated idiopathic granulomatous mastitis: combined intralesional steroid injection with topical steroid administration**. *Breast Care* 2021, **16**(2):181-187.
21. Altintoprak F, Kivilcim T, Yalkin O, Uzunoglu Y, Kahyaoglu Z, Dilek ON: **Topical Steroids Are Effective in the Treatment of Idiopathic Granulomatous Mastitis**. *World J Surg* 2015, **39**(11):2718-2723.
22. Gunduz Y, Altintoprak F, Tatli Ayhan L, Kivilcim T, Celebi F: **Effect of topical steroid treatment on idiopathic granulomatous mastitis: clinical and radiologic evaluation**. *Breast J* 2014, **20**(6):586-591.
23. Toktas O, Toprak N: **Treatment Results of Intralesional Steroid Injection and Topical Steroid Administration in Pregnant Women with Idiopathic Granulomatous Mastitis**. *European Journal of Breast Health* 2021, **17**(3):283.
24. Gurleyik G, Aktekin A, Aker F, Karagulle H, Saglamc A: **Medical and surgical treatment of idiopathic granulomatous lobular mastitis: a benign inflammatory disease mimicking invasive carcinoma**. *Journal of breast cancer* 2012, **15**(1):119-123.
25. Calis H, Karabeyoglu SM: **Follow-up of granulomatous mastitis with monitoring versus surgery**. *Breast Dis* 2017, **37**(2):69-72.
26. Elzahaby IA, Khater A, Fathi A, Hany I, Abdelkhalek M, Gaballah K, Elalfy A, Hamdy O: **Etiologic revelation and outcome of the surgical management of idiopathic granulomatous mastitis; An Egyptian centre experience**. *Breast Disease* 2016, **36**(4):115-122.

Appendix 2.

Complete remission (CR) was defined as the eradication of all the inflammatory signs. including resolution of erythema and lump, or fistula with wound total healing.

Recurrence was meant that the aforementioned sign reemerged after the complete remission.

Treatment	Study	Total patient number	No. of patient in treatment strategy	Complete remission rate(%)	Recurrence rate(%)	Follow-up
Medical						
Antibiotics	Yau (2010) [1]	11	10	0	NA	18.6m
	Neel (2013) [2]	23	14	28.6	21.4	75m(14m-28years)
	Hur (2013) [3]	50	3	33.3	NA	32m
	Akcan (2014) [4]	74	19	0	NA	3m-170m
	Bouton (2015) [5]	37	22	0	NA	7.4m(0-20m)
	Mahlab-Guri (2015) [6]	17	10	20	0	0.5y-11y

	Yabanoglu (2015) [7]	77	8	0	NA	(16-34m)
	Aghajanzadeh (2015) [8]	206	206	2.9	NA	9-18m
	Freenman (2017) [9]	14	8	12.5	NA	3m(2-24m)
	Bashir (2017) [10]	18	15	0	NA	14.1m(3m-36m)
	Tang (2021) [11]	77	43	30.2	11.8	18 m (1-131 m)
	Overall		371	7.2	63.2	
Oral steroids	Sakurai (2011) [12]	8	5	100	0	40.7m(14-67m)
	Neel (2013) [2]	23	2	50	0	75m(14m-28years)
	Hur (2013) [3]	50	13	30.8	NA	32m
	Pandey (2014) [13]	49	44	79.5	22.7	6-12m
	Karanlik (2014) [14]	60	23	100	30.4	22-78m
	Mahlab-Guri (2015) [6]	17	2	50	0	0.5y-11y
	Sheybani (2015) [15]	22	15	66.7	0	11.9m(6-22m)
	Kiyak (2014) [16]	24	1	0	NA	34.8m(10m-66m)
	Yabanoglu (2015) [7]	77	44	86.4	20.5	(16-34m)
	Bashir (2017) [10]	18	3	0	NA	14.1m(3m-36m)
	Tang (2020) [17]	49	3	0	NA	9m(3m-14m)
	Montazer (2020) [18]	30	30	63.3	15.8	10.14 m - 15.12 m
	Tang (2021) [11]	77	15	80	NA	18 m (1-131 m)
	Shojaee (2021) [19]	87	23	100	39.1	26 m (8-48 m)
	Toktas (2021) [20]	78	26	69.2	NA	13.8 m - 32.3 m
	Overall		249	74.3	24.5	
Abx + steroids	Yau (2010) [1]	11	3	0	NA	18.6m
	Sakurai(2011) [12]	8	3	66.7	0	40.7m(14-67m)
	Neel (2013) [2]	23	8	87.5	71	75m(14m-28years)
	Altintoprak (2015) [21]	28	21	0	NA	37.2m(12-72m)
	Mahlab-Guri (2015) [6]	17	4	0	NA	0.5y-11y
	Aghajanzadeh (2015) [8]	206	200	72.2	NA	9-18m

	Freeman (2017) [9]	14	3	3.3	NA	3m(2-24m)
	Bashir (2017) [10]	18	6	100	66.7	14.1m(3m-36m)
	Overall		248	59.5	60	
Steroid + MTX	Neel (2013) [2]	23	2	0	NA	75m(14m-28years)
	Mahlab-Guri (2015) [6]	17	1	0	0	0.5y-11y
	Sheybani (2015) [15]	22	11	81.6	NA	11.9m(6-22m)
	Aghajanzadeh (2015) [8]	206	56	71.4	NA	9-18m
	Freeman (2017) [9]	14	2	50	NA	3m(2-24m)
	Overall		74	67.6	0	
Topical/ local steroid	Gunduz (2014) [22]	11	11	100	18.2	17m(12-48m)
	Altintoprak (2015) [21]	28	28	100	10.7	37.2m(12-72m)
	Tang (2020) [17]	49	12	100	NA	9m(3m-14m)
	Toktas (2021) [20]	78	32	65.6	NA	13.8 m - 32.3 m
	Toktas (2021) [23]	6	6	100	0	19.5 m
	Overall		89	87.6	11.1	
Surgical I&D	Yau (2010) [1]	11	6	100	100	18.6m
	Gurliyik (2012) [24]	19	3	0	NA	20m(6m-75m)
	Neel (2013) [2]	23	6	0	NA	75m(14m-28years)
	Hur (2013) [3]	50	15	33.3	20	32m
	Gunduz (2014) [22]	11	9	0	NA	17m(12-48m)
	Bouton (2015) [5]	37	1	NA	NA	7.4m(0-20m)
	Mahlab-Guri K (2015) [6]	17	4	75	0	0.5y-11y
	Kiyak (2014) [16]	24	9	100	11.1	34.8m(10m-66m)
	Yabanoglu (2015) [7]	77	26	NA	NA	(16-34m)
	Aghajanzadeh (2015) [8]	206	38	0	NA	9-18m
	Calis (2017) [25]	19	3	100	33.3	11m(4m-13m)
	Bashir (2017) [10]	18	9	0	NA	14.1m(3m-36m)
	Overall		129	25.5	34.6	

Excision	Yau (2010) [1]	11	9	66.7	100	18.6m
	Neel (2013) [2]	23	17	23.5	75	75m(14m-28years)
	Pandey (2014) [13]	49	2	100	0	6-12m
	Akcan (2014) [4]	74	6	NA	NA	3-170m
	Bouton (2015) [5]	37	5	100	60	7.4m(0-20m)
	Mahlab-Guri (2015) [6]	17	1	100	0	0.5y-11y
	Yabanoglu (2015) [7]	77	14	NA	NA	16m-34m
	Aghajanzadeh (2015) [8]	206	43	0	NA	9-18m
	Freeman (2017) [9]	14	3	NA	NA	3m(2-24m)
	Calis (2017) [25]	19	1	100	0	11m(4m-13m)
	Bashir (2017) [10]	18	10	NA	NA	14.1m(3m-36m)
	Overall		111	24.4	63.1	
Wide excision	Yau (2011) [1]	11	6	100	33.3	18.6m
	Hur (2013) [3]	50	13	92.3	8	32m
	Akcan (2014) [4]	74	47	NA	NA	3m-170m
	Kiyak (2014) [16]	24	15	100	6.7	34.8m(10m-66m)
	Yabanoglu (2015) [7]	77	31	100	0	(16-34m)
	Elzahaby (2016) [26]	30	30	100	3.3	19m(8-44m)
	Freeman (2017) [9]	14	9	100	11.1	3m(2-24m)
	Bashir (2017) [10]	18	6	100	0	14.1m(3m-36m)
	Tang (2020) [17]	49	9	100	NA	9m(3m-14m)
	Shojaee (2021) [19]	87	17	100	28	26 m (8-48 m)
	Overall		136	98.5	9.7	
Total mastectomy	Yau (2010) [1]	11	1	100	0	18.6m
	Neel (2013) [2]	23	2	100	0	75m(14m-28years)
	Yabanoglu (2015) [7]	77	2	100	0	16m-34m
	Calis (2017) [25]	19	1	100	0	11m(4m-13m)
	Overall		6	100	0	
Oral steroids + surgery						
I&D	Hur (2013) [3]	50	6	50	33	32m

	Mahlab-Guri (2015) [6]	17	4	12.5	0	0.5y-11y
	Shojaee (2021) [19]	87	47	100	19.1	26 m (8-48 m)
	Overall		57	89.5	19.6	
Excision	Gurliyik (2012) [24]	19	19	100	5.3	20m(6m-75m)
	Neel (2013) [2]	23	1	0	NA	75m(14m-28years)
	Akcan (2014) [4]	74	2	100	0	3m-170m
	Overall		22	95	4.8	
Wide excision	Hur (2013) [3]	50	10	80	10	32m
	Pandey (2014) [13]	49	1	100	NA	6-12m
	Akcan (2014) [4]	74	19	100	0	3m-170m
	Karanlik (2014) [14]	60	37	100	0	22-78m
	Aghajanzadeh (2015) [8]	206	11	100	NA	9-18m
	Bashir (2017) [10]	18	1	100	0	14.1m(3m-36m)
	Tang (2020) [17]	49	1	100	NA	9m(3m-14m)
	Montazer (2020) [18]	30	6	100	0	10.14 m - 15.12 m
	Overall		86	97.7	1.4	
Total mastectomy	Freeman (2017) [9]	14	1	100	NA	3m(2m-24m)
	Overall		1	100	NA	

References:

1. Yau FM, Macadam SA, Kuusk U, Nimmo M, Van Laeken N: **The surgical management of granulomatous mastitis**. *Annals of plastic surgery* 2010, **64**(1):9-16.
2. Néel A, Hello M, Cottereau A, Graveleau J, De Faucal P, Costedoat-Chalumeau N, Rondeau-Lutz M, Lavigne C, Chiche L, Hachulla E: **Long-term outcome in idiopathic granulomatous mastitis: a western multicentre study**. *QJM: An International Journal of Medicine* 2013, **106**(5):433-441.
3. Hur SM, Cho DH, Lee SK, Choi M-Y, Bae SY, Koo MY, Kim S, Choe J-H, Kim J-H, Kim JS: **Experience of treatment of patients with granulomatous lobular mastitis**. *Journal of the Korean Surgical Society* 2013, **85**(1):1-6.
4. Akcan A, Öz AB, Dogan S, Akgün H, Akyüz M, Ok E, Gök M, Talih T: **Idiopathic granulomatous mastitis: comparison of wide local excision with or without corticosteroid therapy**. *Breast Care* 2014, **9**(2):111-111.
5. Bouton ME, Jayaram L, O'Neill PJ, Hsu C-H, Komenaka IK: **Management of idiopathic granulomatous mastitis with observation**. *The American Journal of Surgery* 2015, **210**(2):258-262.
6. Mahlab-Guri K, Asher I, Allweis T, Diment J, Sthoeger ZM, Mavor E: **Granulomatous Lobular Mastitis**. *Sat* 2015, **19**:20.
7. Yabanoğlu H, Çolakoğlu T, Belli S, Aytac HO, Bolat FA, Pourbagher A, Tezcaner T, Yildirim S, Haberal M: **A comparative study of conservative versus surgical treatment protocols for 77 patients with idiopathic granulomatous mastitis**. *The Breast Journal* 2015, **21**(4):363-369.
8. Aghajanzadeh M, Hassanzadeh R, Alizadeh Sefat S, Alavi A, Hemmati H, Esmaeili Delshad MS, Emir Alavi C, Rimaz S, Geranmayeh S, Najafi Ashtiani M *et al*: **Granulomatous mastitis: Presentations, diagnosis, treatment and outcome in 206 patients from the north of Iran**. *Breast* 2015, **24**(4):456-460.
9. Freeman C, Xia B, Wilson G, Lewis J, Khan S, Lee S, Lower E, Edwards M, Shaughnessy E: **Idiopathic granulomatous mastitis: a diagnostic and therapeutic challenge**. *The American Journal of Surgery* 2017, **214**(4):701-706.
10. Bashir MU, Ramcharan A, Alothman S, Beaugris S, Khan SA, Sbeih MA, Engdahl R: **The enigma of granulomatous mastitis: A series**. *Breast Dis* 2017, **37**(1):17-20.
11. Tang ELS, Ho CSB, Chan PMY, Chen JJC, Goh MH, Tan EY: **The therapeutic dilemma of idiopathic granulomatous mastitis**. *Ann Acad Med Singap* 2021, **50**(8):598-605.
12. Sakurai K, Fujisaki S, Enomoto K, Amano S, Sugitani M: **Evaluation of follow-up strategies for corticosteroid therapy of idiopathic granulomatous mastitis**. *Surgery today* 2011, **41**(3):333-337.
13. Pandey TS, Mackinnon JC, Bressler L, Millar A, Marcus EE, Ganschow PS: **Idiopathic granulomatous mastitis—a prospective study of 49 women and treatment outcomes with steroid therapy**. *The Breast Journal* 2014, **20**(3):258-266.
14. Karanlik H, Ozgur I, Simsek S, Fathalizadeh A, Tukenmez M, Sahin D, Dursun M, Kurul S: **Can steroids plus surgery become a first-line treatment of idiopathic granulomatous mastitis?** *Breast care* 2014, **9**(5):338-342.
15. Sheybani F, Sarvghad M, Naderi H, Gharib M: **Treatment for and clinical characteristics of granulomatous mastitis**. *Obstetrics & Gynecology* 2015, **125**(4):801-807.
16. Kiyak G, Dumlu EG, Kilinc I, Tokaç M, Akbaba S, Gurer A, Ozkardes AB, Kilic M: **Management of idiopathic granulomatous mastitis: dilemmas in diagnosis and treatment**. *BMC surgery* 2014, **14**(1):1-5.
17. Tang A, Dominguez DA, Edquilang JK, Green AJ, Khoury AL, Godfrey RS: **Granulomatous Mastitis: Comparison of Novel Treatment of Steroid Injection and Current Management**. *J Surg Res* 2020, **254**:300-305.
18. Montazer M, Dadashzadeh M, Moosavi Toomatari SE: **Comparison of the Outcome of Low Dose and High-Dose Corticosteroid in the Treatment of Idiopathic Granulomatous Mastitis**. *Asian Pac J Cancer Prev* 2020, **21**(4):993-996.

19. Shojaee L, Rahmani N, Moradi S, Motamedi A, Godazandeh G: **Idiopathic granulomatous mastitis: challenges of treatment in iranian women**. *BMC Surg* 2021, **21**(1):206.
20. Toktas O, Konca C, Trabulus DC, Soyder A, Koksal H, Karanlik H, Polat AK, Ozbas S, Yormaz S, Isik A: **A novel first-line treatment alternative for noncomplicated idiopathic granulomatous mastitis: combined intralesional steroid injection with topical steroid administration**. *Breast Care* 2021, **16**(2):181-187.
21. Altintoprak F, Kivilcim T, Yalkin O, Uzunoglu Y, Kahyaoglu Z, Dilek ON: **Topical Steroids Are Effective in the Treatment of Idiopathic Granulomatous Mastitis**. *World J Surg* 2015, **39**(11):2718-2723.
22. Gunduz Y, Altintoprak F, Tatli Ayhan L, Kivilcim T, Celebi F: **Effect of topical steroid treatment on idiopathic granulomatous mastitis: clinical and radiologic evaluation**. *Breast J* 2014, **20**(6):586-591.
23. Toktas O, Toprak N: **Treatment Results of Intralesional Steroid Injection and Topical Steroid Administration in Pregnant Women with Idiopathic Granulomatous Mastitis**. *European Journal of Breast Health* 2021, **17**(3):283.
24. Gurleyik G, Aktekin A, Aker F, Karagulle H, Saglamc A: **Medical and surgical treatment of idiopathic granulomatous lobular mastitis: a benign inflammatory disease mimicking invasive carcinoma**. *Journal of breast cancer* 2012, **15**(1):119-123.
25. Calis H, Karabeyoglu SM: **Follow-up of granulomatous mastitis with monitoring versus surgery**. *Breast Dis* 2017, **37**(2):69-72.
26. Elzahaby IA, Khater A, Fathi A, Hany I, Abdelkhalek M, Gaballah K, Elalfy A, Hamdy O: **Etiologic revelation and outcome of the surgical management of idiopathic granulomatous mastitis; An Egyptian centre experience**. *Breast Disease* 2016, **36**(4):115-122.
